# Development of Structured Sunflower Oil Systems for Decreasing Trans and Saturated Fatty Acid Content in Bakery Creams

**DOI:** 10.3390/foods10030505

**Published:** 2021-02-26

**Authors:** María Espert, Teresa Sanz, Ana Salvador

**Affiliations:** Department of Physical and Sensory Properties of Food and Consumer Science, Instituto de Agroquímica y Tecnología de Alimentos (IATA-CSIC), Avda. Agustín Escardino 7, 46980 Paterna, Valencia, Spain; mespert@iata.csic.es (M.E.); tesanz@iata.csic.es (T.S.)

**Keywords:** structured emulsions, fat replacement, bakery cream, cellulose derivatives, physical properties

## Abstract

In this work, the design of low moisture (10%) oil/water emulsions based on sunflower oil were investigated, as well as their application in a bakery cream as a conventional fat replacer. The emulsions were dehydrated to reach 10% moisture content, achieving highly concentrated vegetable oil gel emulsions of different consistencies and qualities. Physical properties of the dried emulsions were evaluated by texture, microstructure, and oil loss determination. The reformulated bakery creams with the dried emulsions obtained from 47% oil showed better spreadability, viscosity, and viscoelasticity properties. A shortening replacement with the dried emulsion obtained from 70% initial oil caused a negative impact on the creams’ consistency, with lower viscosity and lower hysteresis area, revealing a weakness of structure. This research provided new knowledge about the structuration of vegetable oils through concentrated emulsions and their application as a source of healthy fat in creams for bakery applications.

## 1. Introduction

Edible oil structuring has recently gained increasing interest in the field of food because of the possibility of reducing saturated fats with oil rich in unsaturated fatty acids [1]. Strategies to provide solid structure to oils include the organogelation and the design of structured emulsions among others [2,3]. Organogel (referred to as oleogels if the organic phase is an edible oil) can be defined as an organic liquid entrapped within a generally thermo-reversible, anhydrous and structured ‘gel-like’ material by a three-dimensional network [4]. The term “organogelation” has been commonly used in many other types of physical systems that may behave rheologically as a solid (e.g., foams, emulsions, and polymers); however, they should be more appropriately called “hydrophobic soft matter” [1].

Emulsions are generally susceptible to physical instability and tend to breakdown over time and are not able to provide a solid-like texture [5]. However, among the approaches available for structuring oils, it is known that structured emulsions stabilised by amphiphilic molecules have the potential to emerge as an effective method for texture modification in oil structuring. The use of polymers allows the immobilization of liquid oil drops trapped in a structure formed by the gelled water phase, transforming the liquid oil into solid or semi-solid structures which could mimic the physical properties of conventional fats [4,6].

From concentrated oil in water emulsions stabilised by a polymer, an oil gel can be obtained through a dehydration and subsequent shearing process (indirect approach). Nevertheless, partial dehydration of the emulsions will result in a dried intermediate system with greater stability and a higher concentration of oil content than the initial emulsion. Depending on the drying levels and the type of structuring agent used in the emulsion, it will be possible to obtain final systems with different texture patterns and different technological qualities. The mechanical properties of the dried emulsion and its stability could be the determining factors to develop the corresponding structured oleogels by the emulsion-templated approach [7]. Several hydrocolloids (pectin, xanthan gum, cellulose ethers) or a combination of them have been added to the water phase of the emulsions to stabilise the phases and obtain an improvement in the structuring of the oils. One of the advantages of polymeric oil gels is that they are relatively thermally stable [4].

The main objective of this work is focused on obtaining new structured sunflower oil gel systems with 10% moisture content through the use of cellulose ether. The effect of the type and concentration of cellulose ether and the oil concentration in the initial emulsions on the mechanical properties and stability of the dried gel emulsions were investigated. The new dried emulsions will be applied as shortening replacer in a bakery cream through textural and rheological evaluation of the final bakery creams.

## 2. Methodology

### 2.1. Preparation of the Different Systems

#### 2.1.1. Preparation of Initial Emulsions

Emulsions composed of sunflower oil (Deoleo S.A., Córdoba, Spain), cellulose ether (Dow Chemical Co., Brussels, Belgium), and drinking water were prepared. Overall, three types of cellulose ethers were used: two methylcelluloses (MC) with different molecular weight (MX-methycelulose with 95% methoxyl content and A4M-methycelulose with 30% methoxyl content) and one hydroxypropyl methyl cellulose (HPMC) (F4M- 29% methoxyl and 6.8% hydroxypropyl content). In total, two percentages of cellulose ether (0.5 and 1% (*w*/*w*)) and two percentages of sunflower oil (47 and 70% (*w*/*w*)) were studied. The water percentage was adjusted up to 100%. The emulsions were prepared by first dispersing the cellulose ether in the oil using a Heidolph stirrer (RZR 1) (Heidolph Instruments, Germany) at 280 min^−1^ for 5 min. Then, water at 1 °C was gradually added to hydrate the mixture under continuous stirring for 30 s. At last, the mixture was homogenised using a high-energy dispersing unit (Ultraturrax T-18, IKA, Germany) at 6500 rpm for 15 s and at 17,500 rpm for 60 s [8].

#### 2.1.2. Emulsion Drying

Once the emulsion was prepared, a small amount was placed in a silicone mould (6.5 cm diameter and 3 cm thickness) and was dried in a conventional drying oven (Vaciotem-T, JP Selecta, Barcelona (Spain)) at 80 °C for the time necessary to reach a 10% moisture content. A gel consistency is achieved in the obtained dried emulsions. The moisture content was calculated with the following equation:

(1) $$Moisture\;\left(g\right)=\frac{m_{\begin{array}{c} em\;b.d.\;\; \end{array}}\left(g\right)\cdot Water\;\left(\%\right)}{100}-\left(m_{em\;b.d.}\left(g\right)-m_{em\;a.d.}\left(g\right)\right)$$

(2) $$Moisture\;\left(\%\right)=\;\frac{Moisture\;\left(g\right)\cdot 100}{\frac{m_{em\;b.d.}\left(g\right)\cdot Water\left(\%\right)}{100}}$$

where:

*m_em b.d_*_._: Emulsion weight before drying.
*m_em a.d_*_._: Emulsion weight after drying.
*Water* (%): Water content in the emulsion.
*Moisture* (*g*): Emulsion moisture, in grams, after frying.
*Moisture* (%): Emulsion moisture, in %, after drying.

#### 2.1.3. Preparation of Bakery Creams

The ingredients used to prepare the different bakery cream were sugar (30% (*w*/*w*)), milk powder (10% (*w*/*w*)), water (20% (*w*/*w*)), and dried emulsion (30% (*w*/*w*)). In case of control cream, the dried emulsion was replaced by rafined palm fat. The fat replacement was by weight; both creams contained 30% (*w*/*w*) of solid fat or oleogel. 

The preparation of the creams was done on a food processor Thermomix TM31 (Vorwrek, Wuppertal, Germany) and was carried out in two stages. In the first stage, all the ingredients, except the fat (palm fat in the case of the control cream and dried emulsion in the reformulated creams), were mixed at speed 2 for 6 min at a temperature of 50 °C, and then the mixture was left to cool to room temperature. In the second stage, the fat was added and mixed at speed 2 for 5 min. Once the cream was made, it was stored in the refrigerator at 5 °C. All the analyses were carried out 24 h after the preparation of the creams.

The creams formulated with the dried emulsions obtained from 70% oil and 1% F4M could not be measured, since their appearance and texture were not suitable, being very heterogeneous and with presence of lumps.

### 2.2. Properties of Dried Products

#### 2.2.1. Penetration Test

A TA-XT plus Texture Analyzer (Stable Micro Systems, Godalming, UK) was used to measure the texture of the different systems, using the Texture exponent software (Stable Microsystems, Godalming, UK). The dried emulsions were demoulded from the silicon mould (6.5 cm diameter and 3 cm thickness) and the texture properties were determined by means of a penetration test using a 1 cm wide tooth-shaped probe (Volodkevich Bite Jaw-VB). A penetration speed of 1 mm/s and a distance of 10 mm were selected. The parameters of maximum force (N) and the area under the curve (N s) were studied.

#### 2.2.2. Microstructure Evaluation of Dried Emulsion

The evaluation of the microstructure of the dried emulsions was carried out with a Nikon Eclipse E800 V-PS100E optical microscope (Nikon, Tokyo, Japan) using polarised light. A very thin layer of each formulation was placed on a glass slide and observed with the 20× objective, with a working distance of 15 mm. They were observed two hours after drying.

#### 2.2.3. Physical Stability of Dried Emulsion

To study the physical stability of the dried emulsions, the oil loss was evaluated. For this purpose, the cylindrical gels were placed on a sheet of filter paper (Whatman Diameter 110 mm, grade number 1 reference No. 1001-110, Buckinghamshire, UK), and the diameter of the halo formed after two hours was measured.

### 2.3. Physical Properties of the Bakery Creams

#### 2.3.1. Spreadability Test 

Spreadability of creams was determined using a TTC Spreadability Rig (HDP/SR) attachment. Samples were filled into a female cone (90° angle) and were penetrating 22 mm using a corresponding male cone (90° angle) at a speed of 1 mm/s. Maximum force (N), as a measure of firmness, and area under the curve (AUC; N*s), as an index of spreadability, was recorded. 

#### 2.3.2. Rheological Properties of the Bakery Creams

In order to study the viscoelasticity of the different creams, an ARG2 controlled stress rheometer (TA Instruments (Crawley, England)) associated with the TRIOS software was used and all the measurements were made at 20 °C. A 40 mm diameter parallel plate geometry with a rough surface to prevent the sample (40 mm diameter and 1 mm thickness) from slipping during the measurement and a gap of 1 mm were used. At first, a stress sweep was performed between 0.1 to 200 Pa at 1 Hz to determine the lineal viscoelastic region (LVR). Then, frequency sweeps were carried out (10–0.1 Hz) at a fixed stress within the linear zone to determine the viscoelastic behaviour, and both the values of storage modulus (G′), loss modulus (G″), and tan δ were recorded.

The viscosity of the samples was evaluated by rotational flow tests. Up and down flow curves were performed in control shear rate mode from 0.5 to 50 s^−1^ and from 50 to 0.5 s^−1^. Data acquisition was logarithmic taking 10 points per decade and setting a time of 10 s per point.

### 2.4. Statistical Analysis 

The statistical analysis of the data was performed using an analysis of one factor variance (ANOVA) using XLSTAT statistical software (version 2014.5.02, Microsoft Excel^®^, Barcelona, Spain). To determine significant differences, the Tukey Test was used at 95% statistical significance (*p* < 0.05). All tests were performed in triplicate on samples from different days.

## 3. Results and Discussion

### 3.1. Visual Appearance of the Initial Emulsions and Dried Emulsions

**Figure 1:**
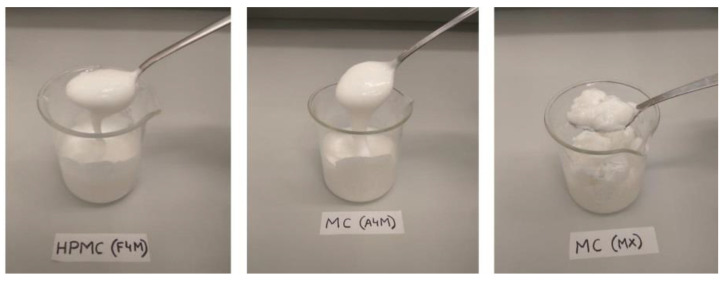
Appearance of initial emulsions prepared with different cellulose ethers.

The appearance of the initial emulsions prepared with the different types of cellulose at 1% is shown in Figure 1, as representative example. The initial emulsion consistency varies considerably depending on the type of cellulose ether used. The emulsion with HPMC (F4M) was the most fluid, compared to the emulsions with MC (A4M and MX); especially MX type. The MX emulsion showed the highest consistency, which is associated to its higher methoxyl content and its higher molecular weight compared to the other cellulose ethers [8]. All starting emulsions had a semi-solid consistency, which easily flows upon the application of a force. The aim of dehydrating the emulsions is to obtain systems with higher plasticity, so they could be used in foods that require plastic fat enlarging the number of food applications.

**Figure 2:**
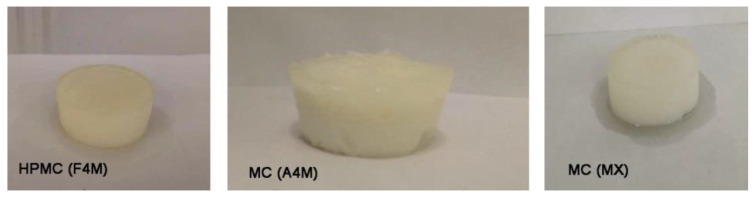
Appearance of dried emulsions containing different cellulose ether.

It is assumed that the formation of a stable concentrated system with a high oil retention capacity will depend on the emulsifying properties of the cellulose ethers and the changes in its structure during the dehydration process. The appearance of the dried emulsions (10% moisture) is shown in Figure 2. All samples had a white structured gel appearance. Depending on the percentage of oil in the initial emulsion (47 or 70%) and the type and percentage (0.5 or 1%) of cellulose ether, the appearance of the dried emulsions varies considerably. MC MX dried emulsion showed a lighter and more ductile texture than the other systems. The following sections specify in more detail how the composition of the dried emulsions influences their physical properties.

### 3.2. Penetration Profile of the Dried Emulsions

**Figure 3:**
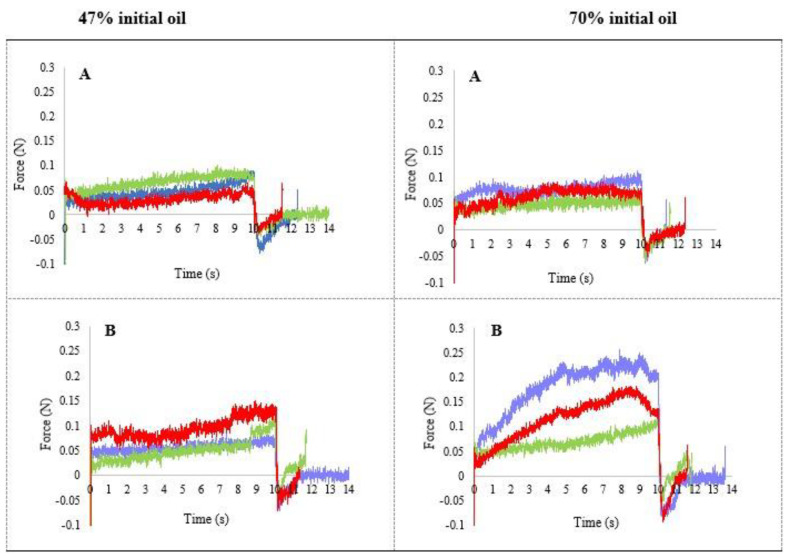
Penetration profile of the dried emulsions with different initial oil content. (**A**): 0.5% cellulose ether; (**B**): 1% cellulose ether. ■ control cream ■ A4M (methylcellulose) ■ F4M (hydroxypropyl methyl cellulose).

The force–time profiles corresponding to the dried emulsions obtained from emulsions with 47 and 70% initial oil content and the different cellulose ether at 0.5 and 1% are presented in Figure 3. The texture profiles reflect the typical structure of soft gels; the force exerted by the probe to penetrate the sample remains constant during the penetration time studied, with no fracture peak observed To objectively compare the texture of the different systems, the area under the curve (AUC) was calculated (Table 1 and Table 2), being considered representative of the general behaviour. 

The softer gels (lower force values and AUC) correspond to those obtained from the emulsion with 0.5% cellulose, although the values depended on the type of cellulose used. Table 1 shows that for 0.5% cellulose concentration, F4M presented significantly the highest AUC, and MX the lowest one. However, as the proportion of cellulose increased (1%) the AUC was significantly higher in MX cellulose, which provided the hardest gels.

An increase in AUC was found in the dried emulsions obtained from emulsions with 70% oil (Figure 3, Table 2). In the 70% oil–1% cellulose curves the force values were higher and not constant during penetration. Although no breakage peak was observed, an initial increase in force was produced, reflecting that these are gels with a different more consistent structure than the one obtained from emulsions with 47% oil. Additionally, a significant increase in the texture parameters was observed by increasing the concentration of cellulose ether from 0.5 to 1% in all cellulose types. 

In summary, at the two concentrations of cellulose ether studied (0.5 and 1%), the gels obtained from emulsions with lower oil content showed lower penetration force and lower AUC values, which indicate a lower consistency than the gels obtained from emulsions with 70% oil. The increase in cellulose concentration significantly increased the area values in the gels from the emulsions with 70% oil. In the gels obtained from emulsions with 47%, the increase in cellulose concentration was only significant for MX cellulose. 

### 3.3. Microstructure of the Dried Emulsions

**Figure 4:**
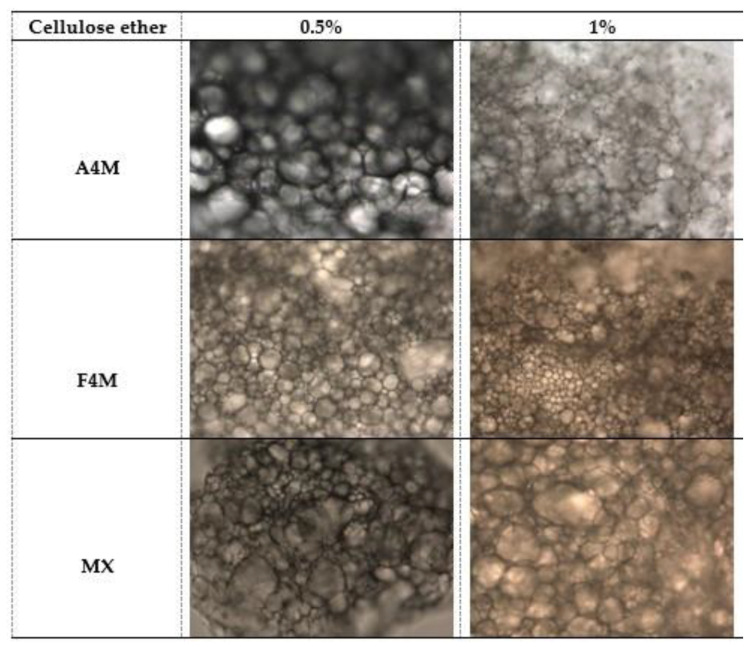
Microscopic observation (20×) of the dried emulsions obtained from 47% sunflower oil emulsions.

**Figure 5:**
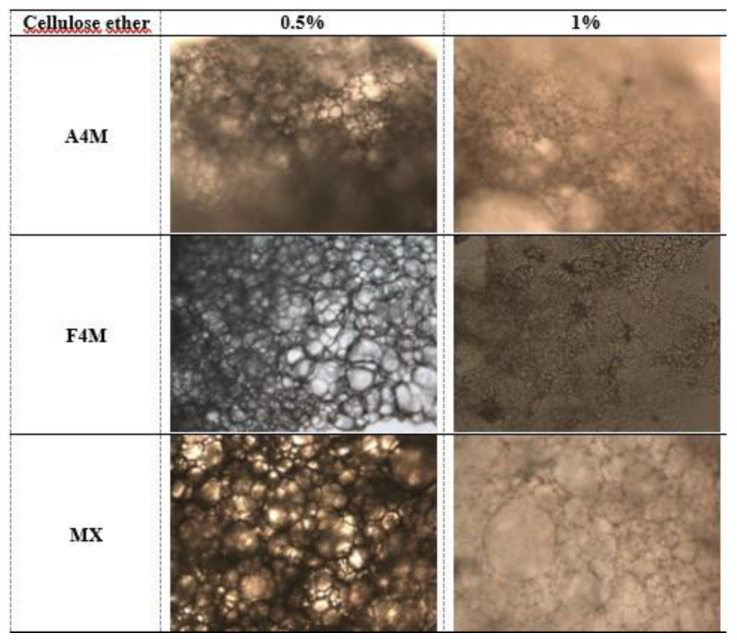
Microscopic observation (20×) of the dried emulsions obtained from 70% sunflower oil emulsions.

The microstructure of the dried emulsions obtained from the different emulsions (47 and 70% oil) is shown in Figure 4 and Figure 5. The microphotographs reveal in all cases a compact matrix in which a high density of oil globules is displayed. The oil droplets are distributed uniformly in the network formed by the functional polymer and the size of the globules varies depending on the type and concentration of cellulose. In case of 47% initial oil emulsion, for the concentration of 0.5%, the largest and the most homogeneous globule size is observed in A4M systems. In MX cellulose the largest globules size and the greatest heterogeneity in size is observed, suggesting that this polymer did not have the required functionality to stabilise the emulsion during the dehydration process [9]. The smallest size and greatest homogeneity is found in F4M cellulose. At the concentration of 1% cellulose a decrease in the size of the oil globules is observed. As well as 0.5% cellulose, the smallest fat globules are observed in F4M cellulose. This fact reveals the greatest effectivity of the interfacial structure provided by F4M to keep the emulsion stable during drying. This result can be explained by the higher affinity for water of this type of cellulose (higher proportion of hydroxypropyl groups), which will provide an improved hydration of the polymer and therefore a more homogeneous structural network to retain the oil after the elimination of water.

In the dried emulsions obtained from 70% initial oil (Figure 5) it can also be appreciated that the increased concentration of cellulose ether forms a considerably more compact and dense microstructure consisting of very small fat globules but with a big level of polidispersity. This fact suggests a positive correlation between hardness and network structure; the dried emulsions with the higher concentration of polymer had the more compact network structure. This phenomenon agrees with the results reported by other authors [10,11]. The smallest size was observed in F4M and A4M cellulose. MX cellulose has the largest size of fat globules and the greatest polydispersion, especially at 1%, showing a clear coalescence of the oil droplets. Coalescence of oil globules on removal of water indicates that this emulsion is not stable to drying. 

### 3.4. Oil Loss of the Dried Emulsions

To quantify the degree of oil retention by the structure of the gel, the different samples were placed on a sheet of filter paper for two hours after the drying process and the diameter of the halo formed was measured. The quality of the gel is closely related to the efficiency of the system to retain the oil in the structural network formed by the polymer.

**Figure 6:**
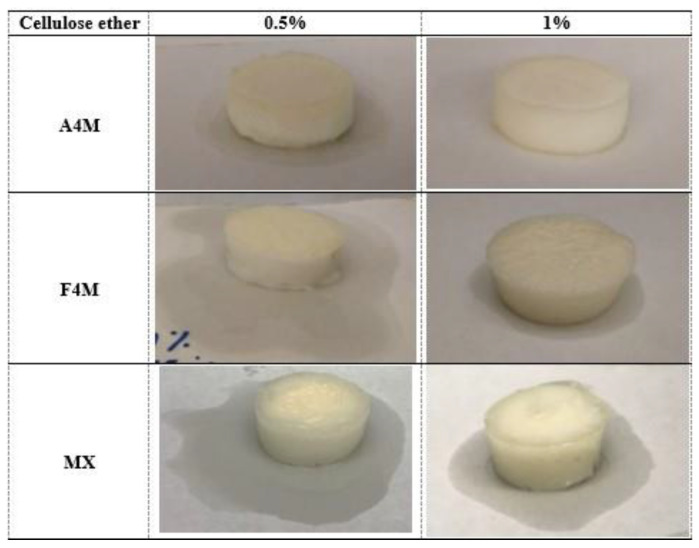
Representation of the qualitative loss of oil (two hours after drying) of the different systems formulated with 47% of oil and different concentration and type of cellulose ether.

**Figure 7:**
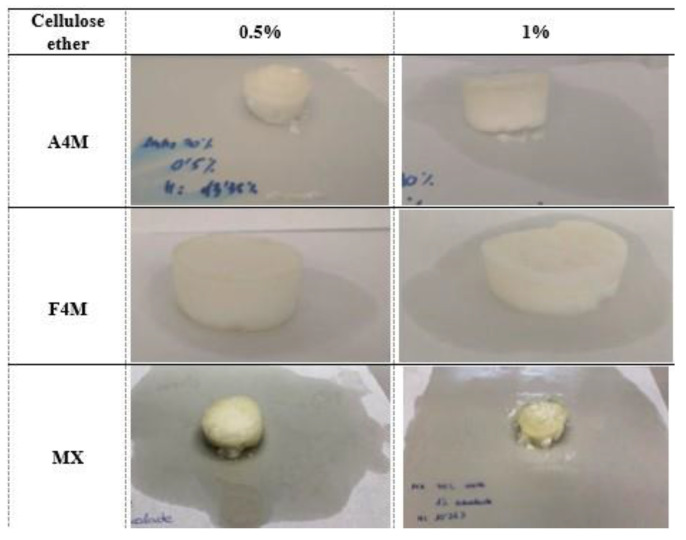
Representation of the qualitative loss of oil (two hours after drying) of the different systems formulated with 70% of oil and different concentration and type of cellulose ether.

Figure 6 and Figure 7 show the photographs of the different systems obtained from emulsions with 47 and 70% oil, respectively, after two hours of drying. At the same concentration of oil and cellulose ether in initial emulsion, the oil loss was higher in MX systems. On the contrary, A4M gels showed the lowest oil loss. At a higher concentration of cellulose ether (1%) a harder consistency was observed, as well as a higher oil binding capacity, regardless of the type of cellulose used. This effect coincides with the decrease in the size of the fat droplets observed when the cellulose concentration increases. The gels obtained from the emulsions with 70% oil showed higher oil loss at both 0.5 and 1% cellulose ether and showed a less compact appearance, especially the MX, which presented a differentiated texture, more heterogeneous, without the ability to retain the shape. This result confirms the presence of coalescence observed in the microstructure observation. The higher methoxylation of this type of cellulose (MX) decreases the gelling temperature and increases the hardness of the gel (higher AUC). This higher gelation would not be favourable for the stability of the emulsion during drying, since the gel structure formed is not capable of adequately retaining the oil.

### 3.5. Physical Properties of the Bakery Creams

To test the food application of the dried emulsions, bakery creams were formulated using the dried emulsions as a shortening alternative and compared with a control cream prepared using palm fat. Only the cellulose types A4M and F4M were employed, discarding MX, because of the poor results obtained in the mechanical properties and stability trials. 

#### 3.5.1. Spreadability of Creams 

**Figure 8:**
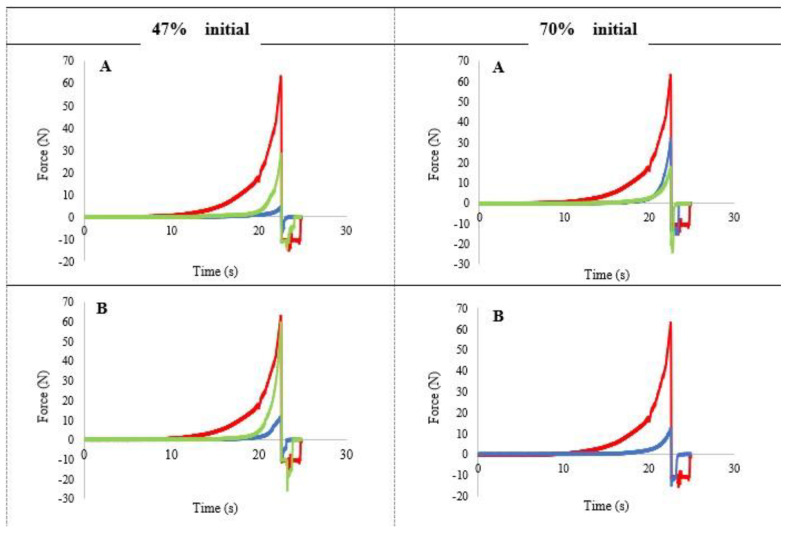
Spreading profile of creams made with the different systems versus control cream. (**A**): 0.5% cellulose ether; (**B**): 1% cellulose ether. ■ control cream ■ A4M ■ F4M.

Spreadability is the ease with which a product can be spread. It is related to the firmness of a product. Figure 8 shows the spreadability curves corresponding to creams formulated with the different dried emulsions as fat substitute compared to control cream. 

Control cream firmness was higher than the reformulated creams, which indicates that palm fat provides a greater firmness or resistance to spread than the dried emulsions. This result was assumable, since the absence of solid fat crystals in sunflower-based emulsions resulted in a lower consistency of the oil structured systems. Previous studies indicated that structured-oil systems provided softer products compared to the use of shortenings or hydrogenated fats [9,12,13]. Bakery creams formulated with 47% oil dried emulsions showed significant differences depending on the type of hydrocolloid used (Table 3), being more spreadable those made with F4M. The cream that most closely resembled the spread profile of the control was the one made with 1% F4M. In contrast, A4M cream was the softest at the two concentrations tested.

Using the dried emulsions with more initial oil (Figure 8 and Table 4), it was observed that the MC A4M cream had an increase in AUC and the maximum peak of firmness. In contrast, F4M reduced these values compared to creams formulated with dried emulsions with less oil. Looking the creams formulated with the 70% oil systems, we can conclude that there were no significant differences regarding the type of cellulose used.

#### 3.5.2. Rheological Properties of Creams 

**Figure 9:**
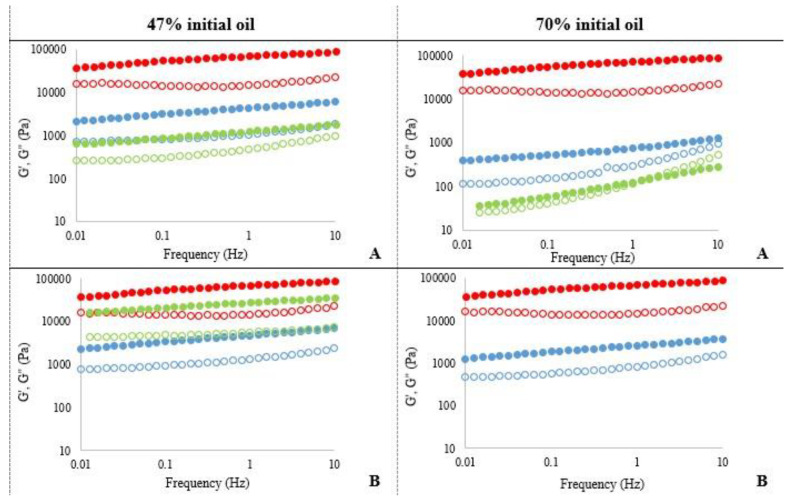
Dynamic viscoelastic properties of the bakery creams as a function of frequency. Storage modulus (G′: filled symbols) and loss modulus (G″: open symbols). (**A**): 0.5% cellulose ether; (**B**): 1% cellulose ether. • control cream • A4M • F4M.

The values of the storage or elastic moduli (G′) and the loss or viscous moduli (G″) as a function of frequency are shown in Figure 9.

The percentage of oil in the initial emulsion clearly affect the cream viscoelastic properties. The creams obtained from the 47% emulsion showed a gel-like behaviour, since the G′ moduli were higher than the G″ moduli across the entire frequency sweep. Besides, a low dependence of moduli on the frequency (0.1–10 Hz) was observed. However, bakery cream formulated with 70% oil and 0.5% F4M showed a clear frequency dependence and the end of the plateau area (crossover of G′ and G″) is visualised, revealing their lower viscoelasticity with an increase in the predominance of the viscous component (G″) versus the elastic (G′), reflecting a more liquid consistency, which is less suitable in terms of spreadable applications where some degree of elasticity is required.

Like the spreadability results, control cream showed the highest viscoelasticity compared to all the reformulated creams. Considering creams made from 47% oil dried emulsion, at 0.5% hydrocolloid, A4M creams showed slightly higher values of G′ and G″ than F4M cream, although differences in viscoelastic properties varying the type of cellulose were not significant (Table 5). However, by increasing the cellulose ratio (1%), F4M cream experienced a marked increase in viscoelasticity.

The shortening replacement with 70% initial oil dried emulsions negatively affected the viscoelasticity of the creams, since it produced a notable decrease in the moduli. The lowest viscoelasticity was found in the F4M cream, in which at 1 Hz G′ and G” moduli are practically matched, confirming the crossing of moduli in the studied frequency spectrum (Table 6). 

**Figure 10:**
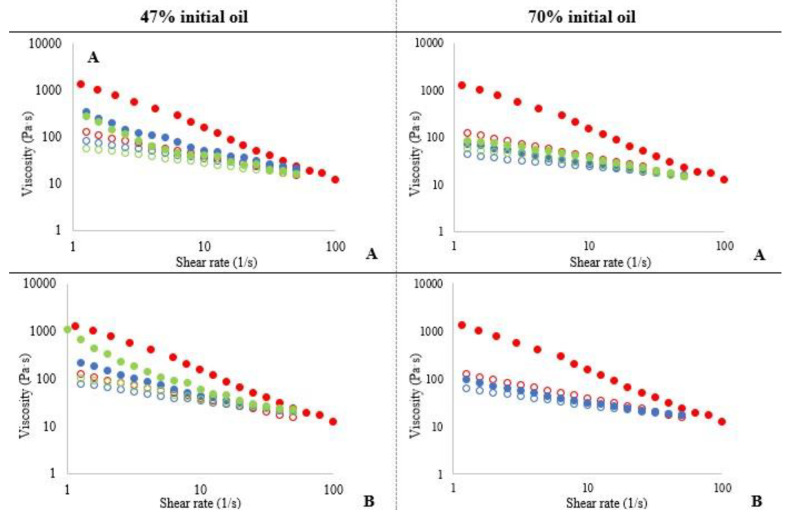
Shear rate-dependent flow behaviour of creams prepared by using the different dried products. (**A**): 0.5% cellulose ether; (**B**): 1% cellulose ether. Upward curves (•, filled symbols); downward curves (∘, open symbols) • control cream • A4M • F4M.

To better understand the creams’ rheological behaviour, flow experiments were also carried out (Figure 10). All creams showed a shear-thinning behaviour, which means that their viscosities decreased progressively as the shear rate increased. This is a consequence of the rearrangement of the molecules into a certain direction under shearing which offered less resistance to flow [14]. Palm fat control cream showed higher apparent viscosity over the range of shear rates compared to the reformulated creams, and a stronger shear-thinning behaviour. These result coincided with those obtained in spreadability and oscillatory trials. The upward viscosity curve is higher than the downward one, especially in the control cream. The hysteresis area among the up and down curves indicates that the sample’s flow is time dependent. The hysteresis area is positively related to the energy required to break the structure. The highest hysteresis area of the control reflects its most complex structure, also reflected by its higher viscoelasticity and spreadability. In creams formulated with 0.5% hydrocolloid systems, both types of cellulose (A4M and F4M) showed similar behaviour. However, by increasing cellulose concentration to 1%, F4M cream presented an increase in viscosity, placing it above the cream with A4M. This change of F4M cream with the increase in the hydrocolloid concentration was also observed in the oscillation and texture determinations. It should be noted that the behaviour of all the creams became more similar at high shear rates, an aspect to consider from a practical point of view, in relation to spreading of the creams. At lower shear rates, there will be clear differences in the consistency of the samples, but these differences will disappear upon increasing shear rate.

Creams formulated with the dried emulsion with 70% initial oil show a lower viscosity and also a lower area of hysteresis, revealing the weakness of their structure. Although dried emulsions exhibited higher texture values when containing higher oil concentration, when applied to creams, a contrary effect is produced: creams formulated from emulsions with 70% initial oil show a drastic decrease in viscosity compared to emulsions starting from 47% initial oil content. The reduced viscosity of the bakery creams formulated from 70% initial oil could be attributed to the lowest oil retention in the dried emulsion matrix, which will be increased by blending during the processing of the cream.

An alternative topic of study with regard to food application of the dried emulsions could be blending them with shortenings. Previous studies have proved that partial replacement of shortening with oleogel provided much more acceptable quality than total replacement in bakery products [15,16].

## 4. Conclusions

Emulsions with less initial oil (47%) provided higher quality systems than emulsions with 70% oil. The dried emulsion obtained from the emulsions with 70% oil showed higher oil loss at both 0.5 and 1% cellulose ether and showed a less compact appearance, especially the MX, which presented a more ductile appearance without the ability to retain the shape, coinciding with a high coalescence of the fat globules. In the case of 47% oil emulsions, the increase in hydrocolloid (1%) did produce an improvement in texture and oil retention, although the MX continued to show low stability. 

The obtained dried emulsions gels were evaluated as shortening replacers in a bakery cream. The creams formulated with the dehydrated emulsions from 47% oil showed better results in spreadability, viscosity, and viscoelasticity. 

The results have demonstrated that depending on the amount of oil and the amount and type of structuring agent used, systems with different physical qualities are obtained, which could be effective in replacing saturated fat in a bakery cream, providing new textures, and healthier fatty acid profile.

## Figures and Tables

**Table 1:** Area under the curve (N s) of dried emulsions obtained from the 47% oil initial emulsion.

	Area Under the Curve (N.s)	
Dried Emulsion Type	Cellulose Ether (%)	
	0.5	1
A4M	0.468 ^bA^	0.508 ^bA^
F4M	0.664 ^aA^	0.512 ^bB^
MX	0.386 ^cA^	0.836 ^aA^

^a,b,c^ Different letters in each column indicate significant differences between the types of cellulose (*p* < 0.05) according to Tukey’s test. ^A,B^ Different letters in each row indicate significant differences between concentration (*p* < 0.05) according to Tukey’s test.

**Table 2:** Area under the curve (N s) of dried emulsions obtained from the 70% oil initial emulsion.

	Area Under the Curve (N.s)	
Dried Emulsion Type	Cellulose Ether (%)	
	0.5	1
A4M	0.573 ^aB^	1.603 ^aA^
F4M	0.475 ^aB^	0.786 ^bA^
MX	0.597 ^aB^	1.021 ^abA^

^a,b,c^ Different letters in each column indicate significant differences between the types of cellulose (*p* < 0.05) according to Tukey’s test. ^A,B^ Different letters in each row indicate significant differences between concentration (*p* < 0.05) according to Tukey’s test.

**Table 3:** Spreadable parameters of creams formulated with the dried emulsion from the 47% oil initial emulsion.

Dried Emulsion Type	Area (N·s)		Max. Force (N)	
	0.5%	1%	0.5%	1%
Control	158.0 ^aA^	158.0 ^aA^	66.0 ^aA^	66.0 ^aA^
A4M	8.3 ^cA^	14.3 ^cA^	5.3 ^cB^	11.8 ^cA^
F4M	34.2 ^bB^	58.4 ^bA^	32.6 ^bB^	59.2 ^bA^

^a,b,c^ Different letters in each column indicate significant differences between the means (*p* < 0.05) according to Tukey’s test. ^A,B^ Different letters in each row indicate significant differences between concentration (*p* < 0.05) according to Tukey’s test.

**Table 4:** Spreadable parameters of creams formulated with the dried emulsion from the 70% oil initial emulsion.

Dried Emulsion Type	Area (N·s)		Max. Force (N)	
	0.5%	1%	0.5%	1%
Control	158.0 ^aA^	158.0 ^aA^	66.0 ^aA^	66.0 ^aA^
A4M	27.8 ^bA^	17.2 ^bB^	25.3 ^abA^	13.2 ^bB^
F4M	22.2 ^b^	-	17.4 ^b^	-

^a,b,c^ Different letters in each column indicate significant differences between the means (*p* < 0.05) according to Tukey’s test. ^A,B^ Different letters in each row indicate significant differences between concentration (*p* < 0.05) according to Tukey’s test.

**Table 5:** Values of G′, G″, and tan δ for creams formulated with the dried emulsion from the 47% oil initial emulsion.

Dried Emulsion Type	0.5%			1%		
	G′	G″	tan δ	G′	G″	tan δ
Control	71,375 ^a^	14,521 ^a^	0.20 ^c^	71,375 ^a^	14,521 ^a^	0.20 ^b^
A4M	5092 ^b^	1321 ^b^	0.26 ^b^	5402 ^c^	1396 ^c^	0.26 ^a^
F4M	1376 ^b^	528 ^b^	0.38 ^a^	21,925 ^b^	4474 ^b^	0.20 ^b^

^a,b,c^ Different letters in each column indicate significant differences between the means (*p* < 0.05) according to Tukey’s test.

**Table 6:** Values of G′, G″, and tan δ for creams formulated with the dried emulsion from the 70% oil initial emulsion.

Dried Emulsion Type	0.5%			1%		
	G′	G″	tan δ	G′	G″	tan δ
Control	71,375 ^a^	14,521 ^a^	0.20 ^c^	71,375 ^a^	14,521 ^a^	0.20 ^b^
A4M	670 ^b^	333 ^b^	0.50 ^b^	2321 ^b^	773 ^b^	0.34 ^a^
F4M	140 ^b^	126 ^b^	0.90 ^a^	-	-	-

^a,b,c^ Different letters in each column indicate significant differences between the means (*p* < 0.05) according to Tukey’s test.
